# Editorial: Evolution of Genetic Mechanisms of Antibiotic Resistance

**DOI:** 10.3389/fgene.2019.00983

**Published:** 2019-10-11

**Authors:** Silvia Buroni, Simona Pollini, Gian Maria Rossolini, Elena Perrin

**Affiliations:** ^1^Department of Biology and Biotechnology, University of Pavia, Italy; ^2^Department of Experimental and Clinical Medicine, University of Florence, Italy; ^3^Microbiology and Virology Unit, Careggi University Hospital, Italy; ^4^Department of Biology, University of Florence, Italy

**Keywords:** antibiotic resistance, evolution, genetic mechanisms, mobile genetic elements, whole genome sequencing

Almost simultaneously with the introduction of antibiotics into clinical practice, antibiotic resistance (AR) emerged as a crucial problem for the treatment of infections (Gould, 2016; Podolsky, 2018). While initially the problem could be solved by new antibiotics, it became relevant with the dearth of the discovery of new classes of molecules (Podolsky, 2018), up to the present days in which, accordingly to World Health Organization (WHO), we are close to a “Post-Antibiotic” era (Reardon, 2014).

While different strategies could be used to try to counteract AR (Lee et al., 2013), a better understanding of the dynamics that lead to its evolution remains essential for the development of more efficient strategies to combat this phenomenon (Davies and Davies, 2010; Lukacisinova and Bollenbach, 2017).

This Research Topic collects 15 articles focused on different aspects of the genetic mechanisms and on the evolution and spread of AR. Starting from the analysis of single genes related to resistance, the topic moves to the analysis of resistance at whole genome and population levels thanks to the recent advances in genome sequencing and analysis, and also covers direct evolution experiments that allowed to follow the emergence, evolution and spread of resistance in defined laboratory conditions.

An in-depth knowledge of the genetic mechanisms of AR allows, for example, to identify new possible antibiotic targets. Two different examples have been reported for mycobacterial species (Machado et al.; Sanz-Garcia et al.). In both cases, characterization of the physiological function of an AR determinant revealed an important role that goes beyond resistance. Sanz-Garcia et al., through a review of the literature on aminoglycoside acetyltransferases in mycobacteria, highlighted the roles of these enzymes in resistance to muramidase enzymes, thanks to their contribution to acetylation of peptidoglycan, and as virulence determinants, since, for example, they control the production of pro-inflammatory cytokines. The authors suggested that these enzymes are promising drug targets rather than a resistance mechanism to aminoglycoside. The work by Machado et al. is instead focused on efflux systems of *Mycobacterium leprae*. The fact that several efflux systems are conserved, despite the drastic genome reduction occurred in this species, indicates a probable role in intracellular survival, making them suitable as possible new antibiotic targets.

Another example of the roles of AR genes in cell biology is reported in the work of Chen et al. They demonstrated that a mutation in the *mprF* gene conferring resistance to daptomycin and vancomycin in methicillin-resistant *Staphylococcus aureus* (MRSA), not only decreases the resistance to oxacillin, but has also a pleiotropic effect on cell membrane and cell wall and on the doubling time.

AR genes can also be used as genetic markers for bacterial population diversity and to monitor AR at different levels. For example, three different alleles of the gene coding for the *norA* efflux pump have been identified in *S. aureus*, and an analysis of the variability of these genes in 112 strains suggests that different variants reflect the population structure of this species (Costa et al.). Three different tetracycline resistance genes together with three tetracyclines molecules have been instead used to monitor the presence of antibiotics and of AR in the longshore sediments of the Three Gorges Reservoir in China (Lu et al.), revealing a significant seasonal variation of both that correlates with a higher use of antibiotics in winter. Finally, Gong et al. developed genetic markers that can be used for population structure analysis and to identify the populations which are most frequently associated with AR.

Beyond single genes involved in AR, it is well known that a fundamental role in the evolution and spread of resistance is played by mobile genetic elements (MGE) that are often associated to more than one mechanism of resistance to different classes of antibiotics. The next-generation and third generation sequencing (long reads sequencing) technologies allowed a better characterization of these elements.

For example, in three different works reported in this research topic, whole genome sequencing (WGS) analysis of different plasmids from clinical isolates allowed a better understanding of the mechanisms of resistance of the associate strains and of the evolution and spread of resistance in hospital environments. In the first article, Paskova et al. reconstructed the important role played by IncX3 plasmids in the dissemination of *bla*_NDM_-like (New Delhi Metallo-Beta-lactamases) genes in both sporadic cases and in an outbreak of NDM-like-producing *Enterobacterales* in Czech hospitals. Di Pilato et al. have instead identified a plasmid lineage that, circulating for over 20 years in different countries in various environmental and clinical *Pseudomonas* spp., contributed to the spread of different Metallo-Beta-lactamases (MBL) encoding genes. Finally, Morroni et al. identified and sequenced a plasmid in *Enterococcus faecium* isolated from a patient in Italy, which is a serious concern since it carries and could co-spread two genes involved in resistance to last resort agents such as oxazolidinones.

Among MGE, also integrative conjugative elements (ICE) have an important role in AR, like for example the Tn*5253* ICE that confers resistance to tetracycline and chloramphenicol in *Streptococcus pneumoniae*. Santoro et al. elucidated the mechanisms of excision and circularization that allowed the transfer of this ICE.

WGS data are useful not only to study MGE associated with AR genes, but also to study the complete resistome of a certain species. A review of the WGS data obtained in different kind of experiments in *Pseudomonas aeruginosa*, for example, highlighted how these data not only allowed to understand the evolutionary dynamics of AR, but are also useful to design specific therapeutic strategies (Lopez-Causape et al.). Similar results have been obtained in a comparative analysis between the genomes of some *Stenotrophomonas acidaminiphila* strains that revealed the evolution of sulfamethoxazole resistance in this species (Huang et al.).

WGS and all the recent -omic technologies have also been the driving force for the development of several direct evolution experiments that allowed to follow and study in a controlled laboratory environment how AR emerges, evolves and spreads. Two examples of these experiments are reported in this research topic. The first one suggested that the development of resistance is in some ways predictable, since *P. aeruginosa* populations evolved in parallel in the presence of antibiotics showed similar patterns of resistance mutations (Sanz-Garcia et al.). Moreover the authors demonstrated that bacteria are able to develop higher levels of resistance to antibiotics to which they are considered intrinsically resistant (Sanz-Garcia et al.). Similar results have been obtained by Lasek-Nesselquist et al. that, using a continuous culture bioreactor model to avoid the problem of nutrients depletion, found that daptomycin resistance in *S. aureus* arises from a combination of mutations localized in few loci, and that most of these mutations appeared early, at low frequency, within the population.

A further step forward in this field is the construction of synthetic bacterial communities to be used for evolution experiments closer to real natural conditions than the use of single strains. An example of the construction and the characterization of a synthetic bacterial community for studies of experimental evolution is reported in the work of Cairns et al.

To conclude, in this Research Topic different aspects of the genetic mechanisms of AR have been addressed. New possible antibiotic targets have been identified and new information on how AR appears and diffuses have been obtained. We think that this aspect of AR is important and need to be further investigated, since trying to avoid the emergence and spread of AR could be essential to face the “Post-Antibiotic” era.

## Author Contributions

EP wrote the manuscript; SB, SP, and GMR participated in manuscript correction.

## Funding

EP was funded by an “Assegno Premiale” from the Department of Biology, University of Florence. SB is supported by the Italian Ministry of Education, University and Research (MIUR): Dipartimenti di Eccellenza Program (2018–2022) − Department of Biology and Biotechnology “L. Spallanzani,” University of Pavia.

## Conflict of Interest

The authors declare that the research was conducted in the absence of any commercial or financial relationships that could be construed as a potential conflict of interest.
